# Job Crafting Mediates the Relationship Between Ego‐Resiliency and Subjective Well‐Being Among Community Nurses in China: A Multicenter Cross‐Sectional Study

**DOI:** 10.1155/jonm/1392815

**Published:** 2026-09-23

**Authors:** Wei Jiang, Jianshu Zhang, Jia Xie, Ronghua Fang

**Affiliations:** ^1^ General Practice Ward, International Medical Center Ward, General Practice Medical Center, West China Hospital, Sichuan University/West China College of Nursing, Sichuan University, Chengdu 610041, China, scu.edu.cn

**Keywords:** community nurse, ego-resiliency, job crafting, mediating effect, subjective well-being

## Abstract

**Aims:**

To examine the association between community nurses’ ego‐resiliency and subjective well‐being (SWB) and investigate the mediating role of job crafting (JC) in this association.

**Background:**

JC is a proactive activity in which nurses adapt job aspects (physical, cognitive, or social) to make them more meaningful. Studies exploring the relationships among nurses’ JC, ego‐resiliency, and SWB are scarce.

**Methods:**

This multicenter cross‐sectional study collected data from 505 registered nurses across 20 community health service centers in Chengdu, Sichuan Province, between May and September 2025 via online questionnaires. The measures included the JC, ego‐resiliency, and SWB questionnaires. Data analysis was conducted using SPSS 26.0 and the PROCESS macro.

**Results:**

The findings revealed that the community nurses’ ego‐resiliency score was 36.68 ± 8.83, their SWB score was 64.71 ± 10.50, and their average JC score was 3.37 ± 0.62. There were positive correlations between JC and ego‐resiliency (*r* = 0.654, *p* < 0.01) as well as SWB (*r* = 0.259, *p* < 0.01) among community nurses. Mediation analysis via the bootstrap method revealed that the mediating effect of JC on the association between ego‐resiliency and SWB was statistically significant, with *β* = 0.068 and 95% CI = [0.018, 0.100]. No zero value was contained in the confidence interval, further verifying the existence of the mediation pathway.

**Conclusion:**

Ego‐resiliency, JC, and SWB were positively associated among community nurses, and JC partially mediated the association between ego‐resiliency and SWB.

**Implications for Nursing Management:**

These results may provide theoretical support and practical implications for further understanding community nurses’ SWB and ego‐resiliency. They also highlight the potential relevance of JC in nursing management and suggest that targeted management strategies may be useful for supporting community nurses’ SWB.

## 1. Introduction

Subjective well‐being (SWB) refers to an individual’s cognitive and emotional assessment of their life, encompassing life satisfaction as well as positive and negative emotions [1]. Positive emotions are generally associated with greater work enthusiasm and efficiency, a stronger sense of belonging, and better work quality and productivity [2]. SWB is closely associated with nurses’ turnover intention [3], work engagement [4], and even patient safety [5] in nursing. A previous study reported that nurses with higher SWB are better able to adapt to high‐pressure work environments and improve overall care quality by reshaping workflows [6].

Although China’s primary healthcare system has undergone rapid expansion, its development has not kept pace with that of the broader health system [7]. Moreover, the share of nurses in this system declined from 22.77% in 2009 to 20.80% in 2018 [8]. In response to accelerating population aging and the nationwide implementation of family physician contracting services, community health institutions have assumed an expanding scope of responsibilities, including preventive care and chronic disease management, thereby intensifying workforce demands [8]. As core members of family doctor teams, community nurses play a pivotal role in coordinating multidisciplinary, patient‐centered care [9]. Nevertheless, nurses confront multiple systemic challenges: chronic understaffing, limited material and technological resources, insufficient compensation, rising clinical and administrative service expectations, and considerable emotional labor demands [10, 11]. These cumulative pressures threaten both the sustainability and quality of community‐based healthcare delivery [12] and are negatively associated with community nurses’ SWB. Therefore, identifying modifiable factors associated with community nurses’ SWB has both theoretical and practical significance.

The present study was guided by the job demands‐resources (JD‐R) model. Within this framework, ego‐resiliency is conceptualized as a personal resource because it reflects an individual’s flexible ability to adapt to stressors, regulate emotions, and mobilize coping strategies under demanding conditions [13]. According to the JD‐R model, work engagement emerges when personal and job resources enable employees to cope with job demands and support personal growth [14], and such engagement can in turn be linked to more positive well‐being outcomes. Nurses with greater ego‐resiliency may be more likely to engage in JC, thereby partly explaining the positive association between ego‐resiliency and SWB [15, 16].

Prior empirical studies have documented bivariate associations among nurses’ JC, psychological resources, and SWB—specifically, between JC and psychological resources and between ego‐resiliency and SWB [14, 17]. However, the current working conditions of community nurses in China remain challenging. Heavy workloads, limited staffing, insufficient public recognition, and high turnover intention can all undermine their occupational identity and SWB. This situation is not conducive to improving community health service capacity [10]. Therefore, greater attention should be given to community nurses’ SWB and psychological resiliency. On the basis of this theoretical rationale, this study proposes the following hypothesis.

• Hypothesis 1: Ego‐resiliency is positively associated with SWB.

Ego‐resiliency signifies an individual’s ability to adapt flexibly and manage conflicts independently, avoiding behavioral or emotional problems even within stressful, adverse, and threatening environments [18]. Individuals with higher ego‐resiliency consistently show a greater ability to buffer against job burnout by developing a positive, optimistic outlook to cope with job stress and recover quickly from adversity [19, 20]. Therefore, higher ego‐resilience levels were associated with fewer depressive symptoms [21]. However, the specific mechanisms underlying the association between ego‐resiliency and SWB remain underexplored. This gap is particularly evident within non‐Western contexts, such as China’s community nursing context, where unique cultural norms and healthcare structures may profoundly shape these dynamics.

• Hypothesis 2: JC is positively correlated with nurses’ SWB.

JC involves self‐initiated changes to job design to better align with psychological needs such as autonomy, mastery, and affiliation. JC can further stimulate nurses’ creativity, personality, and job autonomy, ultimately improving their well‐being, emerging as a potential mediator in this relationship [22]. JC has been measured using instruments such as the Job Crafting (JC) Scale based on the JD‐R model and the Needs‐Based Job Crafting Scale (NJCS). A previous study has suggested that JC is associated with greater work engagement, job satisfaction, and overall well‐being, possibly through greater access to job resources and better management of job demands [23]. An empirical study has demonstrated that JC interventions, including e‐learning programs, can consistently increase work engagement and act as a buffer against stress‐related adverse outcomes such as autonomic nervous system dysregulation [24]. Accordingly, we predict that JC is positively related to SWB.

• Hypothesis 3: Ego‐resiliency is positively associated with JC.

Ego‐resiliency is a strong predictor of work engagement [18]. Research carried out among Chinese nurses has confirmed that JC is associated with higher levels of work commitment and more abundant job resources [25]. These JC behaviors, in turn, were linked to enhanced work engagement and overall well‐being. That is, resilient nurses exhibit proactive behaviors in terms of modifying job demands, which is in line with the JD‐R theory regarding the utilization of personal strengths [26]. This adaptive process may be associated with the fulfillment of psychological needs and lower perceived occupational stressors [27], suggesting that ego‐resiliency may be related to intentional JC efforts in clinical practice.

• Hypothesis 4: JC mediates the relationship between ego‐resiliency and SWB.

Although ego‐resiliency, JC, and SWB have been examined separately in nursing populations, evidence regarding their integrated relationships remains limited, especially among community nurses in China. Existing research has focused predominantly on hospital‐based nurses, or nurses working during public health crises [14, 26]. In contrast, community nurses work in primary health care settings that differ substantially from hospital‐based clinical environments. Whether proactive JC serves as a mediating mechanism between ego‐resiliency and SWB in this population remains unclear. Clarifying this relationship may help explain how psychological resources are associated with well‐being‐related outcomes in community nursing and provide evidence for targeted management interventions. Consequently, this study aimed to investigate whether JC mediates the association between ego‐resiliency and SWB among community nurses in China.

## 2. Methods

### 2.1. Setting and Sample

This multicenter cross‐sectional study was conducted across 20 community health service centers in Sichuan Province, located in southwestern China, from May to September 2025. Convenience sampling was used to recruit eligible community nurses. An online questionnaire link and a quick response (QR) code were distributed through community health center coordinators. This digital method ensured convenience and streamlined data collection. Nurses who met the study’s inclusion and exclusion criteria were enrolled. The inclusion criteria were as follows: (1) informed consent and voluntary participation in this study; (2) nursing staff with a Chinese nurse practitioner certificate; and (3) employment in a community health center. The exclusion criteria were as follows: (1) inability to cooperate in completing the survey and (2) sick leave or maternity leave during the investigation.

According to commonly used sample size estimation principles for multivariable questionnaire‐based studies, the sample size should be at least 5 to 10 times the number of observed variables [28]. In this study, the 68 observed variables referred to questionnaire items and demographic variables collected in the survey, including items from the JC scale, ego‐resiliency scale, SWB scale, and demographic characteristics. Considering a 20% invalid response rate, the required sample size was estimated to be 408–816 participants. A total of 520 questionnaires were distributed. Questionnaires with a response time of less than 30 s were excluded to ensure data quality. Finally, 505 valid questionnaires were obtained, for an effective response rate of 97.11%.

### 2.2. Measures

#### 2.2.1. Demographic and Professional Characteristics

The demographic and professional characteristics encompassed the following variables: gender (male/female), age (in years), educational attainment (technical secondary school, junior college, bachelor’s degree, master’s degree or higher), employment status (within the establishment vs. outside the establishment), marital status (unmarried, married, divorced, widowed), years of nursing experience, professional title (nurse, senior nurse, supervisor nurse, associate senior or above), managerial position (head nurse, teaching instructor, charge nurse, or none), monthly income (in RMB), and perceived job environment satisfaction (assessed using a five‐point Likert scale ranging from “very dissatisfied” to “very satisfied”).

#### 2.2.2. JC Scale

In this study, the Chinese version of the JC scale was used [29]. The scale comprises 21 items across four dimensions: increasing structural job resources (5 items), decreasing hindering job demands (6 items), increasing social job resources (5 items), and increasing challenging job demands (5 items). Each item uses a 5‐point Likert scale (1 = never to 5 = always). The total score ranges from 21 to 105 points, with higher scores indicating a higher level of JC. The Cronbach’s *α* for the overall construct was 0.920 [29]. In this research, the Cronbach’s *α* was 0.919.

#### 2.2.3. Ego‐Resiliency Scale

This study adopted the ego‐resiliency scale revised by Chinese scholars [30]. The scale consists of 14 items rated on a 4‐point Likert scale ranging from 1 (strongly disagree) to 4 (strongly agree). The total score ranges from 14 to 56, with higher scores indicating a higher level of ego‐resiliency. The Cronbach’s *α* coefficient of the original scale is 0.73, and the test‐retest coefficient is 0.712 [30]. In this research, the Cronbach’s *α* was 0.937.

#### 2.2.4. SWB Scale

This study adopted the Chinese version of the SWB scale translated by Duan [31]. The scale encompasses the following 18 items across six dimensions: satisfaction with and interest in life; concern about health; energy levels; melancholic or pleasant mood; control over emotions and behavior; and relaxation and nervousness. The total score ranged from 0 to 120 points. A score ranging from 0 to 24 indicates low SWB, 25 to 48 indicates relatively low SWB, 49 to 72 indicates moderate SWB, 73 to 96 indicates relatively high SWB, and 97 to 120 indicates high SWB. The Cronbach’s *α* coefficient of the scale was 0.878 [31]. In this research, the Cronbach’s *α* was 0.781.

### 2.3. Statistical Analysis

Statistical analysis was conducted using SPSS software (Version 26.0; IBM Corporation, Armonk, NY, USA). The sample distribution was first verified to conform to the normal distribution. Descriptive analyses were used to analyze the demographic characteristics and the study variables. Pearson’s correlation analysis was used to examine the correlations among JC, ego‐resiliency, and SWB. The mediating effect of this study was tested using the bootstrap method from the PROCESS plugin model 4 in SPSS 26.0 [32]. We computed a 95% confidence interval of bias‐corrected percentile bootstrapping through 5000 bootstrap samples. If the 95% confidence interval of the bootstrap does not include 0, it indicates that the mediating effect is significant.

### 2.4. Ethical Considerations

This study was conducted in accordance with the Declaration of Helsinki and was approved by the West China Hospital Ethics Committee (Ethical approval code: No. 2025953). All participants signed informed consent via WeChat‐based signatures.

## 3. Results

### 3.1. Assessment of Common Method Bias

The results of Harman’s single‐factor test revealed 68 factors with eigenvalues greater than 1. The first factor explained 26.23% of the total variance, which was less than the critical criterion of 40% [33]. Therefore, this study did not present severe common method bias.

### 3.2. Demographic Data and Values

Among the 505 participants surveyed (aged 34.71 ± 7.82 years), 97.4% were female and 79.0% were married. In terms of work‐related characteristics, 70.7% had a bachelor’s degree, 73.9% were outside the establishment, 79.0% were married, 60.6% had 10 years or more of working experience, 44.6% were supervisor nurses, 77.6% held no position, 51.5% had earned 3000–4999 yuan/month, and 42.4% were satisfied with their job environment (Table 1).

**TABLE 1:** Demographic characteristics and subjective well‐being scores among community nurses (*n* = 505).

Variables	*N* (%)	Subjective well‐being (M ± SD)	*F*	*p*
Gender			2.469	0.117
Male	13 (2.60)	69.23 ± 8.14		
Female	492 (97.40)	64.60 ± 10.55		
Age, Y			3.243	0.012
20–29	124 (24.60)	66.13 ± 10.56		
30–39	273 (54.10)	63.52 ± 10.74		
40–49	81 (16.00)	65.56 ± 9.76		
50–59	26 (5.10)	66.92 ± 8.09		
≥ 60	1 (0.20)	89.00		
Education level			0.633	0.594
Technical secondary school	11 (2.20)	66.36 ± 11.61		
Junior college	135 (26.70)	65.35 ± 10.64		
Bachelor’s degree	357 (70.70)	64.46 ± 10.46		
Master’s or higher	2 (0.40)	57.50 ± 4.95		
Employment status			0.118	0.732
Within the establishment	132 (26.10)	64.98 ± 9.96		
Outside the establishment	373 (73.90)	64.62 ± 10.72		
Marital status			2.162	0.092
Unmarried	85 (16.80)	67.29 ± 9.61		
Married	399 (79.00)	64.19 ± 10.69		
Divorced	19 (3.80)	63.79 ± 9.66		
Widowed	2 (0.40)	68.00 ± 8.49		
Nursing experience, Y			2.378	0.094
1–5	79 (15.60)	66.72 ± 9.09		
6–9	120 (23.80)	63.41 ± 9.89		
≥ 10	306 (60.60)	64.71 ± 11.03		
Job title			3.081	0.027
Nurse	73 (14.50)	68.04 ± 10.67		
Senior nurse	171 (33.90)	64.61 ± 10.24		
Supervisor nurse	225 (44.60)	63.80 ± 10.76		
Associate senior and above	36 (7.10)	64.19 ± 8.89		
Managerial position			0.887	0.447
Head nurse	90 (17.80)	63.70 ± 9.81		
Teaching instructor	16 (3.20)	64.31 ± 10.76		
Charge nurse	7 (1.40)	60.00 ± 14.26		
None	392 (77.60)	65.05 ± 10.60		
Monthly income (¥)			4.416	0.004
3000–4999	260 (51.50)	64.64 ± 10.54		
5000–6999	168 (33.30)	63.91 ± 10.28		
7000–9999	61 (12.10)	64.85 ± 9.00		
≥ 10,000	16 (3.20)	73.81 ± 14.17		
Job environment satisfaction			17.714	< 0.001
Very dissatisfied	12 (2.40)	60.83 ± 12.54		
Dissatisfied	26 (5.10)	58.00 ± 10.13		
Neutral	193 (38.20)	62.01 ± 9.01		
Satisfied	214 (42.40)	65.95 ± 9.82		
Very satisfied	60 (11.90)	72.70 ± 11.93		

### 3.3. Scores for JC, Ego‐Resiliency, and SWB Among Community Nurses

The average JC score was 3.37 ± 0.62. In the subdomains, the score for increasing structural job resources was 3.85 ± 0.70, the score for increasing social job resources was 3.43 ± 0.72, the score for increasing challenging job demands was 3.22 ± 0.74, and the score for decreasing hindering job demands was 3.04 ± 0.90. The ego‐resiliency score was 36.68 ± 8.83. The SWB score was 64.71 ± 10.50 (Table 2).

**TABLE 2:** The scores of job crafting, ego‐resiliency, and subjective well‐being among community nurses.

Variables	Item	$\overset{¯}{x}\pm \text{s}$
JC	21	3.37 ± 0.62
ISTJR	5	3.85 ± 0.70
ISJR	5	3.43 ± 0.72
ICJD	5	3.22 ± 0.74
DHJD	6	3.04 ± 0.90
Ego‐resiliency	14	36.68 ± 8.83
SWB	18	64.71 ± 10.50

*Note:* ISTJR = increasing structural job resources.

Abbreviations: DHJD, decreasing hindering job demands; ICJD, increasing challenging job demands; ISJR, increasing social job resources; JC, job crafting.

### 3.4. Pearson Correlations Between JC, Ego‐Resiliency, and SWB Among Community Nurses

There were positive correlations between JC and ego‐resiliency (*r* = 0.654, *p* < 0.01) as well as SWB (*r* = 0.259, *p* < 0.01) among community nurses. Ego‐resiliency is significantly positively correlated with SWB (*r* = 0.313, *p* < 0.01) (Table 3).

**TABLE 3:** Pearson correlations between job crafting, ego‐resiliency, and subjective well‐being among community nurses.

Variables	JC	Ego‐resiliency	SWB
JC	1		
Ego‐resiliency	0.654^∗∗^	1	
SWB	0.259^∗∗^	0.313^∗∗^	1

^∗∗^ *p* < 0.01.

### 3.5. Mediating Effect of JC on the Relationship Between Ego‐Resiliency and SWB

**FIGURE 1:**
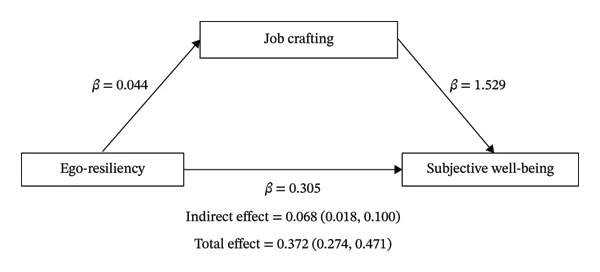
Mediating effect of job crafting on the relationship between ego‐resiliency and subjective well‐being.

A bootstrapping mediation analysis (Process plugin model 4) with 5000 bias‐corrected bootstrap resamples was performed to examine whether JC mediates the relationship between ego‐resiliency and SWB. The total effect of ego‐resiliency on SWB was significant (total effect *β* = 0.372, 95% CI = [0.274, 0.471], *p* < 0.001). The direct effect of ego‐resiliency on SWB, controlling for JC, was also significant (direct effect *β* = 0.305, 95% CI = [0.195, 0.414], *p* < 0.001). The indirect effect was statistically significant (*β* = 0.068, 95% CI = [0.018, 0.100], *p* = 0.006), as the 95% confidence interval did not contain zero. These findings indicate that JC partially mediates the association between ego‐resiliency and SWB. The mediating effect accounted for approximately 18.28% of the total effect (Table 4). The mediating role of JC is shown in Figure 1.

**TABLE 4:** Mediating effect of job crafting on the relationship between ego‐resiliency and subjective well‐being.

Path	Effect *β*	SE	*p*	95% CI	Ratio to the total effect
Ego‐resiliency ⟶ JC	0.044	0.004	< 0.001	[0.036, 0.052]	—
JC ⟶ SWB	1.529	0.559	0.006	[0.431, 2.626]	—
Ego‐resiliency ⟶ SWB (Total effect)	0.372	0.05	< 0.001	[0.274, 0.471]	—
Ego‐resiliency ⟶ SWB (Direct effect)	0.305	0.056	< 0.001	[0.195, 0.414]	—
Ego‐resiliency ⟶ JC ⟶ SWB (Indirect effect)	0.068	0.021	0.006	[0.018, 0.100]	18.28%

Abbreviations: CI, confidence interval; SE, standard error.

## 4. Discussion

This study investigated the associations among ego‐resiliency, JC, and SWB among community nurses and identified JC as a partial mediator. Grounded in the JD‐R model’s resource‐gain perspective, the findings indicate that ego‐resiliency—a stable personal resource—was directly associated with SWB and indirectly associated with SWB through JC, thereby supporting nurses’ adaptive alignment of job demands and resources [15, 34].

The moderate SWB level observed among community nurses was comparable to findings from Saudi Arabia [12] and South Africa [35]. These findings indicate that in resource‐constrained settings or economically less developed settings, limited organizational resources, heavy workloads, insufficient staffing, and limited professional support may place greater pressure on community nurses and primary care professionals, thereby influencing their SWB [10]. In China, community nurses perform multiple tasks in primary health care and family doctor contract services. A heavy, diverse workload causes emotional consumption [9], whereas social stigma and professional status inequality harm professional fulfillment and mental health [10] and increase turnover intention [3]; adequate support and role recognition help maintain well‐being.

The ego‐resiliency level among community nurses was comparable to that reported in a Singaporean [36] but higher than that reported for some clinical nurses in Poland (35.41 ± 5.51) [37]. This discrepancy may stem from differences in study populations, assessment tools, and cultural contexts. In China, community nurses are responsible for patient care in outpatient clinics, wards, and at home, and participate in the daily management of family doctor services. Low salaries, staff shortages, and severe moral distress exacerbate occupational stress, may be associated with emotional exhaustion, deplete psychological resources, and ultimately reduce ego‐resiliency [38].

In this study, the mean JC score of community nurses was moderate to high. These scores were lower than the scores reported by nurses in Saudi Arabia and Spain [39, 40], but higher than those reported by nurses in Egypt [41]. However, these comparisons should be interpreted cautiously, given the differences in measurement instruments, study populations, and healthcare contexts across studies. Among the four dimensions, increasing structural job resources had the highest mean score, whereas decreasing hindering job demands had the lowest score. These findings are consistent with those of Rodríguez‐García et al. [40], suggesting that nursing leaders should recognize the importance of decreasing hindering job demands. However, compared with hospital nurses, community nurses may have fewer formalized career development pathways. Moreover, they may encounter issues such as heavy workloads [42] and insufficient JC‐related training [16] that may restrict their ability to actively adjust their job demands and resources. These are often shaped by the work system rather than individual choice. In contrast, improving competence and work adaptability may be a more feasible strategy for community nurses than reducing hindering job demands. Thus, increasing structural job resources may represent a more realistic and practical form of JC in primary care settings.

Our data showed that ego‐resiliency is positively correlated with SWB (Hypothesis 1 is supported). Nurses with higher levels of resiliency experience greater overall well‐being. In stressful, fatiguing, anxiety‐ and burnout‐inducing work environments, higher ego‐resiliency may be associated with a greater ability to identify positive aspects of work, adopt proactive coping strategies, and maintain better mental health and SWB [43].

JC is positively correlated with SWB (Hypothesis 2 is supported) in this study, which is consistent with existing research findings [22]. From the JD‐R perspective, nurses who seek resources, adjust tasks, reshape relationships, or reduce hindering demands may experience a better balance between job demands and available resources. These behaviors may be related to greater autonomy, meaning, and perceived control at work, which are important psychological conditions associated with SWB.

Furthermore, in this study, JC is significantly positively correlated with ego‐resiliency (Hypothesis 3 is supported). The strong positive correlation between ego‐resiliency and JC suggests that resilient nurses may be more likely to use proactive strategies to manage work demands. Ego‐resiliency may provide the psychological flexibility needed to identify modifiable aspects of work, seek support, and adjust task boundaries [43]. These findings are consistent with the JD‐R assumption that personal resources can facilitate proactive resource‐seeking behaviors [44].

Our study revealed that community nurses’ JC is positively correlated with their SWB. When ego‐resiliency is entered simultaneously, JC partially mediates the association between ego‐resiliency and SWB (Hypothesis 4 is supported). This means that proactive work redesign may be one mechanism linking personal resources and well‐being [37]. This aligns with the findings of other scholars that JC mediates the relationship between ego‐resiliency‐related resources (e.g., workplace justice) and well‐being outcomes [6, 45]. Nevertheless, the mediated proportion was 18.28%, suggesting that the indirect effect was statistically significant but modest in magnitude in this study. These findings imply that JC is meaningful but not sufficient on its own; other pathways, such as coping style [46], social support [47], organizational justice [48], and work–family balance [49], may also explain the relationship between ego‐resiliency and SWB.

Overall, these findings deepen the theoretical interpretation of the JD‐R model. Ego‐resiliency may help community nurses preserve psychological resources under pressure, whereas JC may translate these resources into concrete behavioral adjustments. That is, JC may act as a mechanism through which ego‐resiliency may contribute to improve SWB outcomes such as work engagement, job satisfaction, and reduced burnout [6, 23].

## 5. Limitations of the Study

Several limitations need to be noted in this study. First, its cross‐sectional design limits causal inference. Future studies can examine the dynamic causal relationship and the joint conditioning effect of JC on the association between ego‐resiliency and SWB through longitudinal designs. Second, all the variables are measured via self‐report questionnaires collected at the same time from the same participants, making the study vulnerable to social desirability effects. Future studies can be carried out from multiple perspectives, such as through qualitative or mixed methods. Third, all participants were recruited from 20 community health centers in Sichuan Province without central‐level adjustments, limiting the representativeness of the results. In the future, multicenter, multiregional, and even transnational study designs can be adopted, and stratified or random sampling can be applied whenever possible to improve the representativeness of the findings.

## 6. Conclusion

This study revealed that ego‐resiliency, JC, and SWB were positively associated among community nurses and that JC partially mediated the association between ego‐resiliency and SWB. The findings suggest that proactive work redesign may be an important but modest mechanism through which personal psychological resources are linked to well‐being. Management strategies aimed at supporting JC and ego‐resiliency may be considered as potential approaches to improving community nurses’ SWB, although longitudinal and intervention studies are needed to confirm these effects.

## 7. Implications for Nursing Management

These findings have several practical implications for nursing management. First, future community health institutions should regularly conduct JC scale assessments for nurses and develop structured JC intervention programs to guide nurses in adjusting work requirements, accessing resources, reordering task priorities, and redefining routine community nursing care [34]. Second, nursing managers can establish mentoring systems in which experienced community nurses provide guidance on workload management, family doctor team communication, and emotionally demanding patient interaction coping. Third, nursing administrators should increase feasible job autonomy, for example, by allowing nurses to participate in scheduling, chronic disease follow‐up planning, health education design, and quality‐improvement activities. Finally, ego‐resiliency training programs that include cognitive–behavioral skills, stress regulation, reflective practice, and peer support may help strengthen personal resources. In addition, supportive leadership should be fostered through regular feedback, recognition, fair workload allocation, and open communication channels. These strategies may make JC more feasible and may be associated with better SWB, job satisfaction, and workforce retention.

## Author Contributions

Wei Jiang: questionnaire design, writing–original draft, data preprocessing, and data curation. Jianshu Zhang: questionnaire review, writing–original draft, methodology, and data analysis. Jia Xie: ethical application. Ronghua Fang: resources, supervision, project administration, and revision and refining of the content of the manuscript.

## Funding

No funding was received for this study.

## Conflicts of Interest

The authors declare no conflicts of interest.

## Data Availability

The datasets used during the current study are available from the corresponding author upon reasonable request.
